# Effects of *Enteromorpha prolifera* polysaccharides on growth performance, intestinal barrier function and cecal microbiota in yellow-feathered broilers under heat stress

**DOI:** 10.1186/s40104-023-00932-2

**Published:** 2023-10-10

**Authors:** Wenchao Liu, Huimei Liu, Yaoyao Wang, Zhongxiang Zhao, Balamuralikrishnan Balasubramanian, Rajesh Jha

**Affiliations:** 1 Department of Animal Science, College of Coastal Agricultural Sciences, Guangdong Ocean University, Zhanjiang, Guangdong 524088 People’s Republic of China; 2 Department of Food Science and Biotechnology, College of Life Science, Sejong University, Seoul, 05006 South Korea; 3 Department of Human Nutrition, Food and Animal Sciences, College of Tropical Agriculture and Human Resources, University of Hawaii at Manoa, Honolulu, HI 96822 USA

**Keywords:** Broilers, Cecal microbiota, Heat stress, Intestinal barrier function, Seaweed polysaccharides

## Abstract

**Background:**

Global warming leading to heat stress (HS) is becoming a major challenge for broiler production. This study aimed to explore the protective effects of seaweed (*Enteromorpha prolifera*) polysaccharides (EPS) on the intestinal barrier function, microbial ecology, and performance of broilers under HS. A total of 144 yellow-feathered broilers (male, 56 days old) with 682.59 ± 7.38 g were randomly assigned to 3 groups: 1) TN (thermal neutral zone, 23.6 ± 1.8 °C), 2) HS (heat stress, 33.2 ± 1.5 °C for 10 h/d), and 3) HSE (HS + 0.1% EPS). Each group contained 6 replicates with 8 broilers per replicate. The study was conducted for 4 weeks; feed intake and body weights were measured at the end of weeks 2 and 4. At the end of the feeding trial, small intestine samples were collected for histomorphology, antioxidant, secretory immunoglobulin A (sIgA) content, apoptosis, gene and protein expression analysis; cecal contents were also collected for microbiota analysis based on 16S rDNA sequencing.

**Results:**

Dietary EPS promoted the average daily gain (ADG) of broilers during 3–4 weeks of HS (*P* < 0.05). At the end of HS on broilers, the activity of total superoxide dismutase (T-SOD), glutathione S-transferase (GST), and the content of sIgA in jejunum were improved by EPS supplementation (*P* < 0.05). Besides, dietary EPS reduced the epithelial cell apoptosis of jejunum and ileum in heat-stressed broilers (*P* < 0.05). Addition of EPS in HS group broilers' diet upregulated the relative mRNA expression of *Occludin*, *ZO-1, γ-GCLc* and *IL-10* of the jejunum (*P* < 0.05), whereas downregulated the relative mRNA expression of *NF-κB p65, TNF-α* and *IL-1β* of the jejunum (*P* < 0.05). Dietary EPS increased the protein expression of Occludin and ZO-1, whereas it reduced the protein expression of NF-κB p65 and MLCK (*P* < 0.01) and tended to decrease the protein expression of TNF-α (*P* = 0.094) in heat-stressed broilers. Furthermore, the proportions of *Bacteroides* and *Oscillospira* among the three groups were positively associated with jejunal apoptosis and pro-inflammatory cytokine expression (*P* < 0.05) and negatively correlated with jejunal Occludin level (*P* < 0.05). However, the proportions of *Lactobacillus, Barnesiella, Subdoligranulum, Megasphaera, Collinsella,* and *Blautia* among the three groups were positively related to ADG (*P* < 0.05).

**Conclusions:**

EPS can be used as a feed additive in yellow-feathered broilers. It effectively improves growth performance and alleviates HS-induced intestinal injury by relieving inflammatory damage and improving the tight junction proteins expression. These beneficial effects may be related to inhibiting NF-κB/MLCK signaling pathway activation and regulation of cecal microbiota.

**Supplementary Information:**

The online version contains supplementary material available at 10.1186/s40104-023-00932-2.

## Introduction

Broiler production is the most promising livestock industry to provide a source of high-quality protein for humans. As commercial chicken production has met the market demand, yellow-feathered broilers are becoming increasingly popular among Chinese consumers because of their excellent meat quality. In China, the farming of yellow-feathered broilers is mainly concentrated in South China [1]. However, South China is located in tropical and subtropical regions, and heat stress (HS) becomes a major challenge for producing yellow-feathered broilers [2]. It has been reported that HS induces physiological and metabolic dysfunction, impairs intestinal barrier function by causing oxidative stress and inflammatory response, and disrupts the intestinal microbiota, thus reducing the growth rate in broilers [3]. Studies have shown that nutritional interventions can alleviate HS-induced intestinal barrier damage, thereby improving the thermal tolerance and growth performance of broilers [4]. 

Natural polysaccharides with health benefits have received extensive attention in animal nutrition [5]. Seaweeds contain large amounts of polysaccharides, which have been documented to possess multiple bioactive activities, including antioxidant, antitumor, anticoagulant, and immunomodulatory effects [6]. *Enteromorpha prolifera* belongs to marine green algae and is widely distributed in coastal areas worldwide, especially in China [7]. *E. prolifera,* a traditional edible alga in East Asia, is widely used also as a drug in which polysaccharides are the main functional components [8]. Several studies in broilers have confirmed the antioxidant activity, lipid metabolism and immune regulation, and intestinal health-promoting roles of *E. prolifera* polysaccharides (EPS) [9–11]. Besides, our recent studies demonstrated that dietary EPS could mitigate HS-induced immune organ and duodenal damage of broilers. This effect may be related to the regulation of nuclear factor-erythroid2-related factor 2 (Nrf2) mediated antioxidant and/or nuclear factor-kappaB p65 (NF-κB p65) mediated immune signaling pathway by EPS [12–14]. However, it is still unknown whether EPS can promote the intestinal mechanical barrier function and microbiota balance in broilers challenged with HS.

Therefore, based on the previous studies on the biological activity of EPS and its putative beneficial roles in broilers, this study aimed to evaluate the protective effects of EPS on the intestinal mechanical barrier and cecal microbiota in heat-stressed broilers. We also explored some relevant mechanisms of action, thereby providing a theoretical basis for using EPS as a novel feed additive to mitigate HS in yellow-feathered broiler production.

## Materials and methods

### Experimental birds and diets

The EPS was obtained from Haida Biotechnology Co., Ltd. (Qingdao, Shandong, China). The chemical and monosaccharides composition of EPS is presented in Additional file 1: Table S1. The extraction method and molecular weight of EPS were as described previously [14]. A total of 144 male yellow-feathered broilers (8 weeks old, a local slow-growing broiler breed in Guangdong, China) were used in this study. The selection of chickens was based on our previous studies [1, 2]. Broilers with an initial average body weight (BW) of 682.59 ± 7.38 g were randomly and equally allocated to one of three groups: TN (thermal neutral zone), HS, and HSE (HS + 0.1% EPS) group. Each group had six replicate cages with eight broilers per cage, and the duration of the feeding trial was four weeks. Broilers in the TN group were raised under 23.6 ± 1.8 °C during the whole study period. Broilers in HS and HSE groups were raised under 33.2 ± 1.5 °C for 10 h/d (from 8:00 to 18:00) during the entire study period. The ambient temperature of HS and HSE groups at other times was the same as the TN group. The relative humidity was 55%–75% for all three groups. The broilers had ad libitum access to feed and water. The feeds were formulated to meet the Chinese Chicken Feeding Standard (NY/T33-2004) [15]. The formulation and nutrient composition of the basal diets are presented in Additional file 1: Table S2. The feed provided to broilers was in mash form, and the addition of EPS replaced the equivalent amount of corn in the feed. EPS products were first mixed with 1 kg of wheat bran then mixed with the feed to get a homogenous mix. During the feeding trial, a daily lighting program of 18 h of light and 6 h of darkness was used (18L:6D) for the yellow-feathered broilers [16].

### Growth performance

The BW and feed intake (FI) of broilers were recorded in each cage at the end of week 2 and 4. The BW and FI data were used to calculate the average daily gain (ADG), the average daily feed intake (ADFI), and the feed conversion rate (FCR).

### Sampling procedures

At the end of the feeding trial, one broiler from each cage (*n* = 6 per group) was randomly selected for slaughter by bleeding the neck. Jejunum and ileum were separated immediately after slaughter, and about 1 cm of the intestinal section from the middle of the jejunum and ileum was collected. Samples were fixed into 4% paraformaldehyde for subsequent tissue section preparation, histomorphological observation, apoptosis detection, and immunofluorescence assay. The remaining jejunum and ileum samples were cut, and the intestinal digesta were flushed with PBS solution at 4 °C. About 2 g of jejunum and ileum mucosa samples at the midpoint of the intestinal segment were scraped using a glass slide, stored in liquid nitrogen for pre-freezing, and transferred to a −80 °C refrigerator for subsequent biochemical and molecular analysis. In addition, 1 g of cecal digesta samples from each bird were collected for microbiota analysis.

### Intestinal histomorphology, antioxidant capacity, and sIgA content

The jejunum and ileum samples fixed with paraformaldehyde were embedded, sliced, and stained with hematoxylin and eosin (H&E). The histo-morphological changes of stained samples were observed and photographed under a 40× light microscope. Three visual fields were selected for each slice, and villus height (VH), villus width (VW), and crypt depth (CD) were measured; villus height to crypt depth ratio (VH:CD) was also calculated.

The jejunum and ileum mucosa samples were homogenized, and the antioxidant parameters were determined using commercial kits (Nanjing Jiancheng Bioengineering Institute, Jiangsu, China). The detected antioxidant parameters included the total anti-oxidation capacity (T-AOC) and malondialdehyde (MDA) level, and the enzyme activity of the catalase (CAT), total superoxide dismutase (T-SOD), glutathione-S transferase (GST) and glutathione peroxidase (GSH-Px). The catalog number, specification, and detection methods of the above antioxidant kits are presented in our previous study [12]. In addition, the secretory immunoglobulin A (sIgA) contents in jejunum and ileum mucosa samples were analyzed according to the instructions of sIgA ELISA kit (H108-2, 96T, Nanjing Jiancheng Bioengineering Institute, Jiangsu, China). The protein concentration of intestinal mucosa samples was determined using a commercial kit (A045-3-2, 96T, Nanjing Jiancheng Bioengineering Institute, Jiangsu, China). The results were expressed as antioxidant enzyme activity units and the content of MDA and sIgA per mg protein in the jejunum and ileum mucosa.

### Intestinal apoptosis and immunofluorescence measurements

The paraformaldehyde-fixed jejunum and ileum segments were embedded in paraffin and sectioned for TUNEL apoptosis detection and immunofluorescence in situ protein expression analysis of Occludin and ZO-1. The detailed analysis methods of TUNEL apoptosis and immunofluorescence have been described in our previous study [13]. The reagents and antibody information used in this section are as follows: TUNEL detection kit (Roche, 11684817910, Switzerland); DAPI (Servicebio, G1012, Wuhan, China); fluorescent labeled primary antibodies contain the rabbit polyclonal antibody against Occludin (GB111401, 1:200 dilution, Servicebio, Wuhan, China), and the rabbit polyclonal antibody against ZO-1 (21773-1-AP, 1:100 dilution, Proteintech, Wuhan, China). The secondary antibody is goat polyclonal secondary antibody to rabbit IgG (GB25303, 1:400 dilution, Alexa Fluor® 488, Servicebio, Wuhan, China). Ultimately, the pictures of apoptosis and immunofluorescence were collected using an upright fluorescence microscope (Nikon Eclipse, C1, Japan). The apoptosis-positive cells and positive expression of Occludin and ZO-1 were marked with green color and analyzed using Image-Pro Plus 6.0 software (Media Cybernetics, Inc., Rockville, USA) under 100× magnification. Three visual fields were selected from each slice for apoptosis and immunofluorescence analysis. The apoptosis rate was calculated by the ratio of positive cells to the total cells in each visual field. The positive expressions of Occludin and ZO-1 were calculated as the area density value: the ratio of the accumulated optical density (IOD) of the expression of Occludin and ZO-1 to the area of pixels in each visual field.

### Relative mRNA expression analysis

The total RNA of the jejunum and ileum mucosa samples was extracted using the commercial RNA extraction kit (N066, Nanjing Jiancheng Bioengineering Institute, Jiangsu, China). The quality of the extracted total RNA was detected using 260/280 nm of ultraviolet spectrophotometer. The RNA samples with a ratio of 1.8–2.0 under 260/280 nm were used for reverse transcription (RT) of cDNA with the commercial RT kit (Takara Biotechnology Co., Ltd., Beijing, China). The real-time quantitative PCR (qPCR) was used to measure relative mRNA expression following the reactions and conditions presented in our previous study [12]. The used internal reference gene was *β-actin*, and the primers information is presented in Additional file 1: Table S3. The 2^−ΔΔCt^ method was used for qPCR data processing, and the gene expression is expressed as relative mRNA expression to the TN group.

### Western blot analysis

Western blot was used to assay the protein expression of jejunal mucosa as described previously [12]. The antibodies information is as follows: primary antibody against TNF-α (tumor necrosis factor-α) was from Proteintech Biotechnology Co., Ltd. (Wuhan, China; 17590-1-AP, 26 kDa, 1:2,000 dilution, rabbit polyclonal antibody); primary antibody against NF-κB p65 (nuclear factor-kappaB p65) was from Servicebio Technology Co., Ltd. (Wuhan, China; GB11997, 65 kDa, 1:1,000 dilution, rabbit polyclonal antibody); primary antibody against MLCK (myosinlightchainkinase) was from Abcam Technology Co., Ltd. (Cambridge, UK; ab76092, 211 kDa, 1:1,000 dilution, rabbit monoclonal antibody); primary antibody against Occludin was from Servicebio Technology Co., Ltd. (Wuhan, China; GB111401, 59 kDa, 1:1,000 dilution, rabbit polyclonal antibody); primary antibody against ZO-1 was from Proteintech Technology Co., Ltd. (Wuhan, China; 21773-1-AP, 195 kDa, 1:1,000 dilution, rabbit polyclonal antibody); primary antibody against β-actin was from Servicebio Technology Co., Ltd. (Wuhan, China; GB12001, 42 kDa, 1:1,000 dilution, mouse polyclonal antibody); secondary antibody against IgG was Servicebio Technology Co., Ltd. (Wuhan, China; GB23303, 1:3,000 dilution, HRP-labeled goat anti-rabbit polyclonal antibody). The β-actin was used as internal control, and the results of protein level are presented as target protein/β-actin.

### Cecal microbiota composition

Total genomic DNA samples of cecal digesta were extracted using a commercial kit (51306, QIAGEN, Hamburg, Germany). The PCR amplification of the V3–V4 region of the 16S rDNA gene of the cecal microbiota was performed using the forward primer 338F (5'-ACTCCTACGGGAGGCAGCA-3') and the reverse primer 806R (5'-GGACTACHVGGGTWTCTAAT-3'), and the 16S rDNA gene amplicon sequencing was performed using the Illumina TruSeq Nano DNA LT Library Prep at Suzhou PANOMIX Biomedical Tech Co., Ltd. (Suzhou, China). The bioinformatics analysis of the sequencing data was performed using QIIME2 (2019.4) and R packages (v3.2.0) with slight modifications based on the official tutorials (https://docs.qiime2.org/2019.4/tutorials/). The detailed sequencing and analysis methods were presented in our previous study [17].

### Statistical analysis

The general linear model procedure of SAS 9.4 (SAS Institute Inc., Cary, NC, USA) was performed for the growth performance and intestinal barrier function-related data analysis, and Tukey’s test was used to compare the significance differences in growth performance and intestinal barrier function between TN, HS, and HSE groups. The alpha diversity indexes of cecal microbiota were calculated according to the method described by Thukral [18]; and the significance of differences in alpha diversity between groups was performed by Tukey's test. The detection of intergroup distance difference of microbiota composition was based on distance matrix analysis and the significance of distance differences between groups was tested using the Anosim algorithm [19]. The principal component analysis (PCA) method of cecal microbiota composition was based on the report by Ramette [20]. Orthogonal partial least squares discriminant analysis (OPLS-DA) method of cecal microbiota composition was according to the report by Mahadevan [21]. Tukey's test was used to determine the difference in cecal bacterial proportions between groups. The Spearman analysis was performed to analyze the correlation between the proportions of cecal microbiota and growth performance and intestinal barrier function.* P* < 0.05 was set as the criterion of significance difference.

## Results

### Growth performance and intestinal histomorphology

The effects of EPS on growth performance in heat-stressed broilers are presented in Table 1. During 1–2, 3–4, and 1–4 weeks, broilers in HS and HSE groups had a lower ADFI (*P* < 0.05) than the TN group. During 3–4 weeks, HS led to a reduction in ADG (*P* < 0.05), and dietary supplementation of EPS increased the ADG (*P* < 0.05) of heat-stressed broilers. Also, broilers challenged with HS had a lower ADG during the total study period (*P* < 0.05).

**Table 1:** Effects of *Enteromorpha prolifera* polysaccharides on growth performance of yellow-feathered broilers under heat stress

Items	TN	HS	HSE	SEM	*P*-value
1–2 weeks					
ADG	22.78	21.40	21.85	0.55	0.243
ADFI	79.57^a^	73.90^b^	75.96^ab^	1.43	0.052
FCR	3.50	3.45	3.48	0.04	0.808
3–4 weeks					
ADG	23.92^a^	21.22^b^	22.80^a^	0.43	0.004
ADFI	81.80^a^	74.92^b^	77.98^b^	1.10	0.003
FCR	3.42	3.54	3.41	0.04	0.148
1–4 weeks					
ADG	23.38^a^	21.35^b^	22.36^ab^	0.35	0.008
ADFI	80.71^a^	74.40^b^	76.97^b^	0.99	0.004
FCR	3.46	3.49	3.45	0.03	0.523

*TN* Thermal neutral zone, *HS* Heat stress, *HSE* Heat stress group supplemented 0.1% *Enteromorpha prolifera* polysaccharides, *SEM* Standard error of the mean, *ADG* Average daily gain, *ADFI* Average daily feed intake, *FCR* Feed conversion rate (the ratio of feed/gain), *P, P* value for analysis of variance (ANOVA)

^a,b^Different superscripts in the same row indicate a significant difference between groups (*P* < 0.05)

As shown in Table 2, HS reduced the VH of jejunum and VH:CD of the ileum of broilers (*P* < 0.05). After supplementing EPS, VW, CD, and VH:CD of jejunum and ileum in heat-exposed broilers (both HS and HSE groups) showed no significant difference with the TN group (*P* > 0.05). VH of jejunum and VH:CD of ileum in HS broilers showed a tendency for lower values, while those values were not different from the TN group (*P* < 0.10) for HSE-fed broilers.

**Table 2:** Effects of *Enteromorpha prolifera* polysaccharides on intestinal histomorphology of yellow-feathered broilers under heat stress

Items	TN	HS	HSE	SEM	*P-*value
Jejunum					
VH	1,282.28^a^	1,061.61^b^	1,152.44^ab^	60.92	0.079
VW	236.04	241.32	269.15	17.04	0.373
CD	325.89	353.5	349.56	25.22	0.713
VH:CD	4.07	3.04	3.44	0.38	0.204
Ileum					
VH	1067.72	894.89	906.88	68.00	0.184
VW	197.15	219.10	206.59	13.92	0.554
CD	208.61	305.67	236.78	42.57	0.297
VH:CD	5.38^a^	3.33^b^	4.03^ab^	0.56	0.074

*TN* Thermal neutral zone, *HS* Heat stress, *HSE* Heat stress group supplemented 0.1% *Enteromorpha prolifera* polysaccharides, *SEM* Standard error of the mean, *VH* Villus height, *VW* Villus width, *CD* Crypt depth, *VH:CD* The ratio of villus height/crypt depth, *P P*-value for analysis of variance (ANOVA)

^a,b^Different superscripts in the same row indicate a tendency for a significant difference between groups (*P* < 0.10)

### Antioxidant capacity and sIgA level of jejunum and ileum

As presented in Table 3, broilers under HS had lower enzyme activity of T-AOC, T-SOD, CAT, and GST, and the level of sIgA in the jejunum than those in the TN group (*P* < 0.05). Compared with the HS group, dietary EPS supplementation improved the enzyme activity of T-SOD and GST and the content of sIgA in the jejunum (*P* < 0.05). As shown in Table 4, compared with the TN group, broilers in HS and HSE groups had a reduced T-AOC of the ileum (*P* < 0.05); and HS decreased the sIgA level of ileum in broilers (*P* < 0.05). Meanwhile, the ileal sIgA level of heat-exposed broilers supplemented with EPS had no significant difference from the TN group (*P* > 0.10).

**Table 3:** Effects of *Enteromorpha prolifera* polysaccharides on antioxidant capacity and sIgA level of jejunal mucosa in yellow-feathered broilers under heat stress

Items	TN	HS	HSE	SEM	*P-*value
T-AOC, mmol/mg protein	64.92^a^	25.87^b^	42.37^ab^	7.12	0.043
T-SOD, U/mg protein	321.06^a^	286.85^b^	319.58^a^	7.69	0.070
GSH-Px, U/mg protein	85.72	85.05	89.70	4.36	0.736
CAT, U/mg protein	3.88^a^	1.69^b^	2.67^b^	0.30	0.017
GST, U/mg protein	31.95^a^	19.45^b^	29.68^a^	2.50	0.048
MDA, nmol/mg protein	1.96	3.23	2.09	0.38	0.135
sIgA, ng/mg protein	58.94^a^	45.62^b^	61.33^a^	2.47	0.016

*TN* Thermal neutral zone, *HS* Heat stress, *HSE* Heat stress group supplemented 0.1% *Enteromorpha prolifera* polysaccharides, *SEM* Standard error of the mean, *T-AOC* Total antioxidant capacity, *T-SOD* Total superoxide dismutase, *GSH-Px* Glutathione peroxidase, *CAT* Catalase, *GST* Glutathione S-transferase, *MDA* Malondialdehyde, *sIgA* Secretory immunoglobulin A, *P P* value for analysis of variance (ANOVA)

^a,b^Different superscripts in the same row indicate a significant difference between groups (*P* < 0.05)

**Table 4:** Effects of *Enteromorpha prolifera* polysaccharides on antioxidant capacity and sIgA level of ileal mucosa in yellow-feathered broilers under heat stress

Items	TN	HS	HSE	SEM	*P-*value
T-AOC, mmol/mg protein	57.70^a^	41.03^b^	37.42^b^	3.22	0.022
T-SOD, U/mg protein	295.41	315.60	282.52	26.04	0.689
GSH-Px, U/mg protein	78.83	73.12	86.57	17.86	0.871
CAT, U/mg protein	2.43	3.66	1.85	0.79	0.352
GST, U/mg protein	15.50	17.28	15.30	2.19	0.753
MDA, nmol/mg protein	2.08	2.58	2.46	0.17	0.219
sIgA, ng/mg protein	57.68^a^	48.11^b^	52.90^ab^	2.28	0.016

*TN* Thermal neutral zone, *HS* Heat stress, *HSE* Heat stress group supplemented 0.1% *Enteromorpha prolifera* polysaccharides, *SEM* Standard error of the mean, *T-AOC* Total antioxidant capacity, *T-SOD* Total superoxide dismutase, *GSH-Px* Glutathione peroxidase, *CAT* Catalase, *GST* Glutathione S-transferase, *MDA* Malondialdehyde, *sIgA* Secretory immunoglobulin A, *P P* value for analysis of variance (ANOVA)

^a,b^Different superscripts in the same row indicate a significant difference between groups (*P* < 0.05)

### Apoptosis and relative mRNA expression of jejunum and ileum

**Fig. 1:**
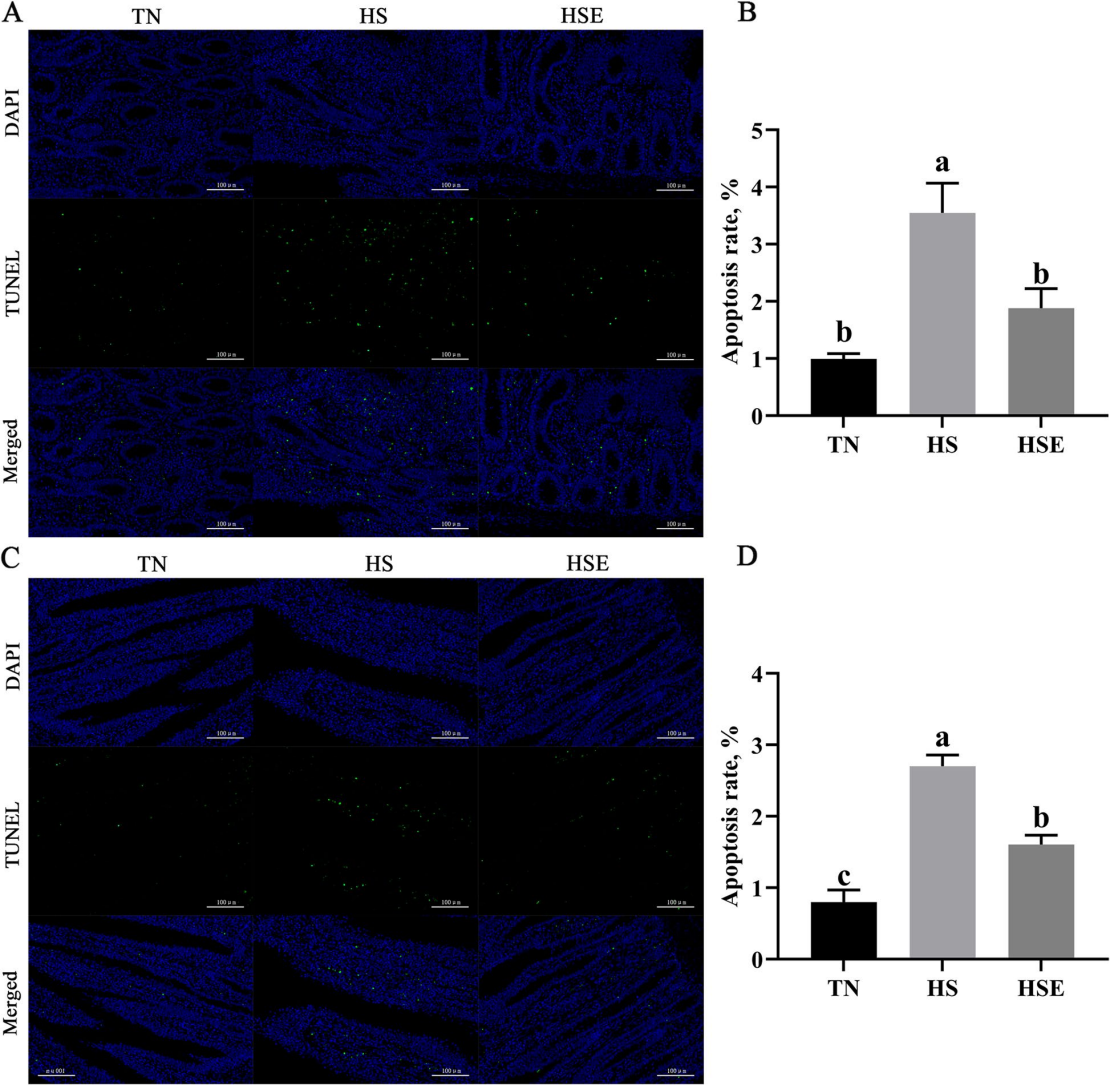
Effects of *Enteromorpha prolifera* polysaccharides on the apoptosis rate of jejunal (**A**, **B**) and ileal (**C**, **D**) epithelial cells in yellow-feathered broilers under heat stress. *TN* Thermal neutral zone, *HS* Heat stress, *HSE* Heat stress group supplemented 0.1% *Enteromorpha prolifera* polysaccharides. **A** and **C**, 200× magnification, the scale bars are 100 μm. ^a−c^Different superscripts indicate a significant difference between groups (*P* < 0.05)

As illustrated in Fig. 1, broilers in the HS group had higher jejunal and ileal apoptosis rates than those in the TN group (*P* < 0.05). However, the apoptosis rate of the jejunum and ileum was decreased by dietary EPS supplementation (*P* < 0.05).

**Fig. 2:**
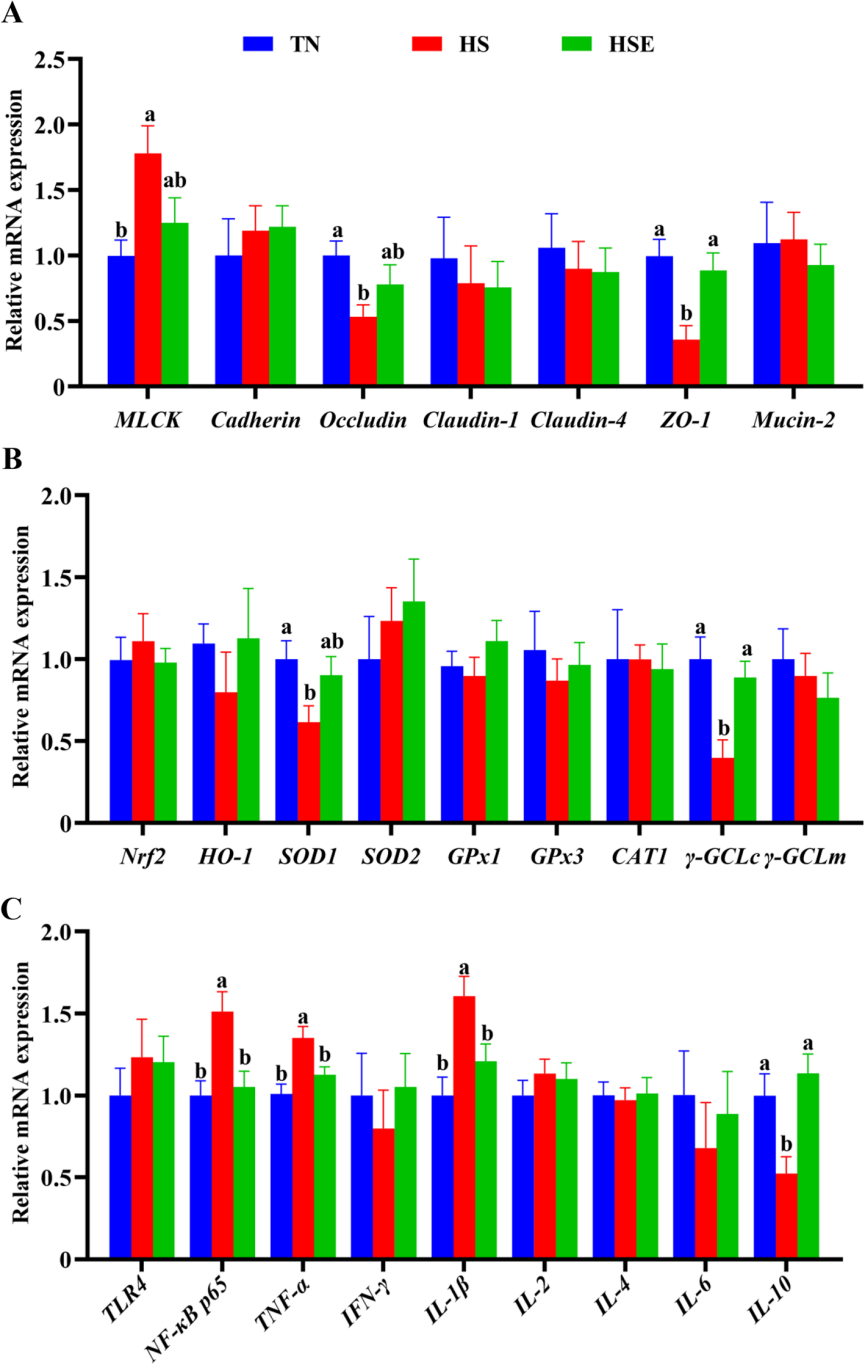
Effects of *Enteromorpha prolifera* polysaccharides on the relative mRNA expression of barrier function (**A**), antioxidant (**B**) and immune (**C**) related genes of jejunum in yellow-feathered broilers under heat stress. *TN* Thermal neutral zone; *HS* Heat stress, *HSE* Heat stress group supplemented 0.1% *Enteromorpha prolifera* polysaccharides. ^a,b^Different superscripts indicate a significant difference between groups (*P* < 0.05)

The relative mRNA expression results of jejunum are shown in Fig. 2. Compared with the TN group, HS upregulated the relative mRNA expression of *MLCK, NF-κB p65, TNF-α* and *IL-1β* (*P* < 0.05); but downregulated the relative mRNA expression of *Occludin, ZO-1, SOD1, γ-GCLc* and *IL-10* of the jejunum (*P* < 0.05). Broilers fed the EPS-supplemented diet showed no significant difference with the TN control group in the measured expression parameters (*P* > 0.05).

**Fig. 3:**
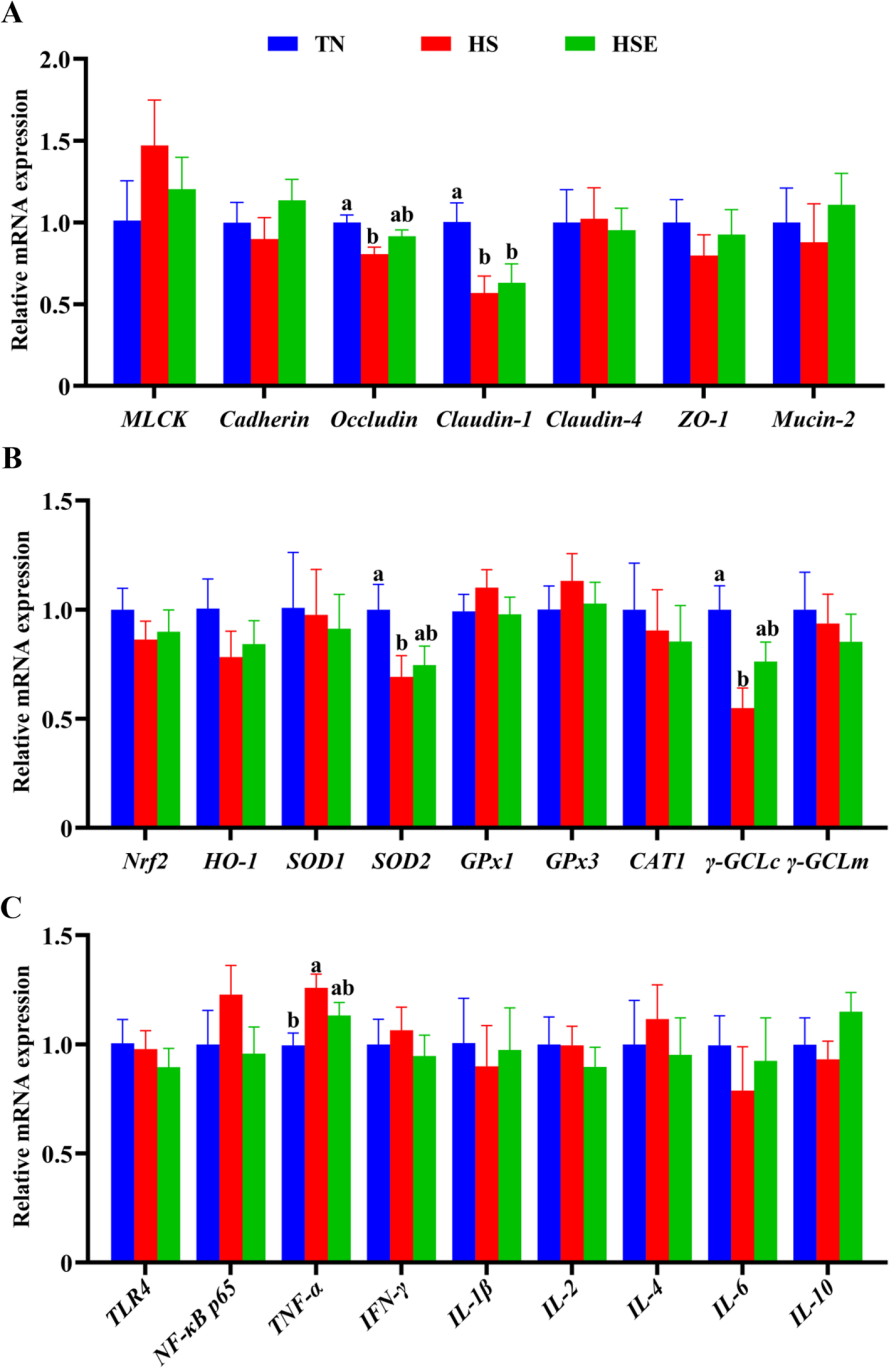
Effects of *Enteromorpha prolifera* polysaccharides on the relative mRNA expression of barrier function (**A**), antioxidant (**B**) and immune (**C**) related genes of ileum in yellow-feathered broilers under heat stress. *TN* Thermal neutral zone, *HS* Heat stress, *HSE* Heat stress group supplemented 0.1% *Enteromorpha prolifera* polysaccharides. ^a,b^Different superscripts indicate a significant difference between groups (*P* < 0.05)

As shown in Fig. 3, broilers in the HS group had lower relative mRNA expression levels of *Occludin, Claudin-1, SOD2,* and *γ-GCLc* (*P* < 0.05); but had higher relative mRNA expression levels of *TNF-α* of ileum than those in TN group (*P* < 0.05). Only the relative mRNA expression of *Claudin-1* was significantly lower than TN values for the HSE group (*P* < 0.05).

### Immunofluorescence analysis of tight junction proteins in jejunum and ileum

**Fig. 4:**
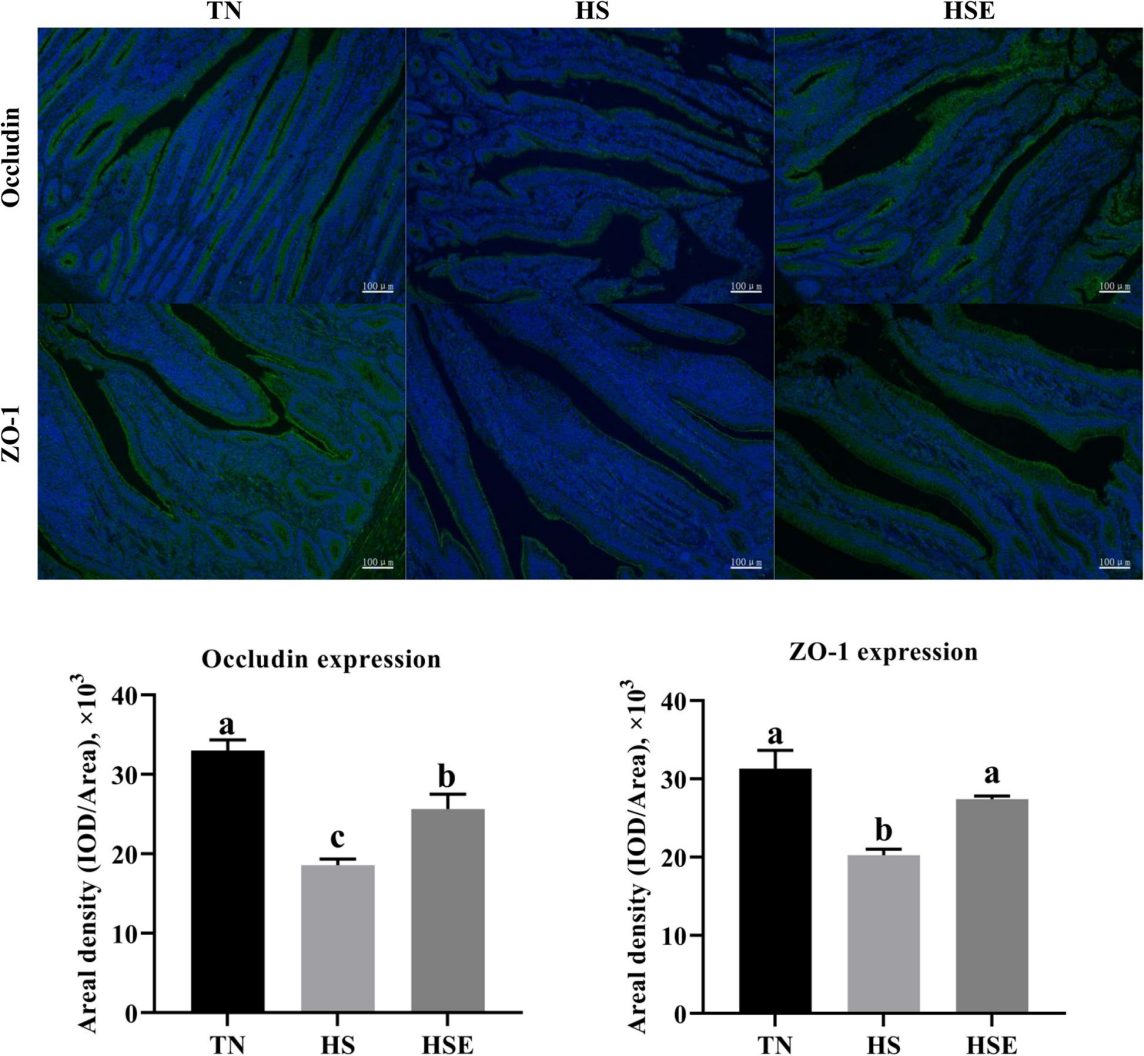
Immunofluorescence analysis of jejunal tight junction proteins in yellow-feathered broilers under heat stress and supplemented with *Enteromorpha prolifera* polysaccharides. *TN* Thermal neutral zone, *HS* Heat stress, *HSE* Heat stress group supplemented 0.1% *Enteromorpha prolifera* polysaccharides. 100× magnification, the scale bars are 100 μm. ^a−c^Different superscripts indicate a significant difference between groups (*P* < 0.05)

**Fig. 5:**
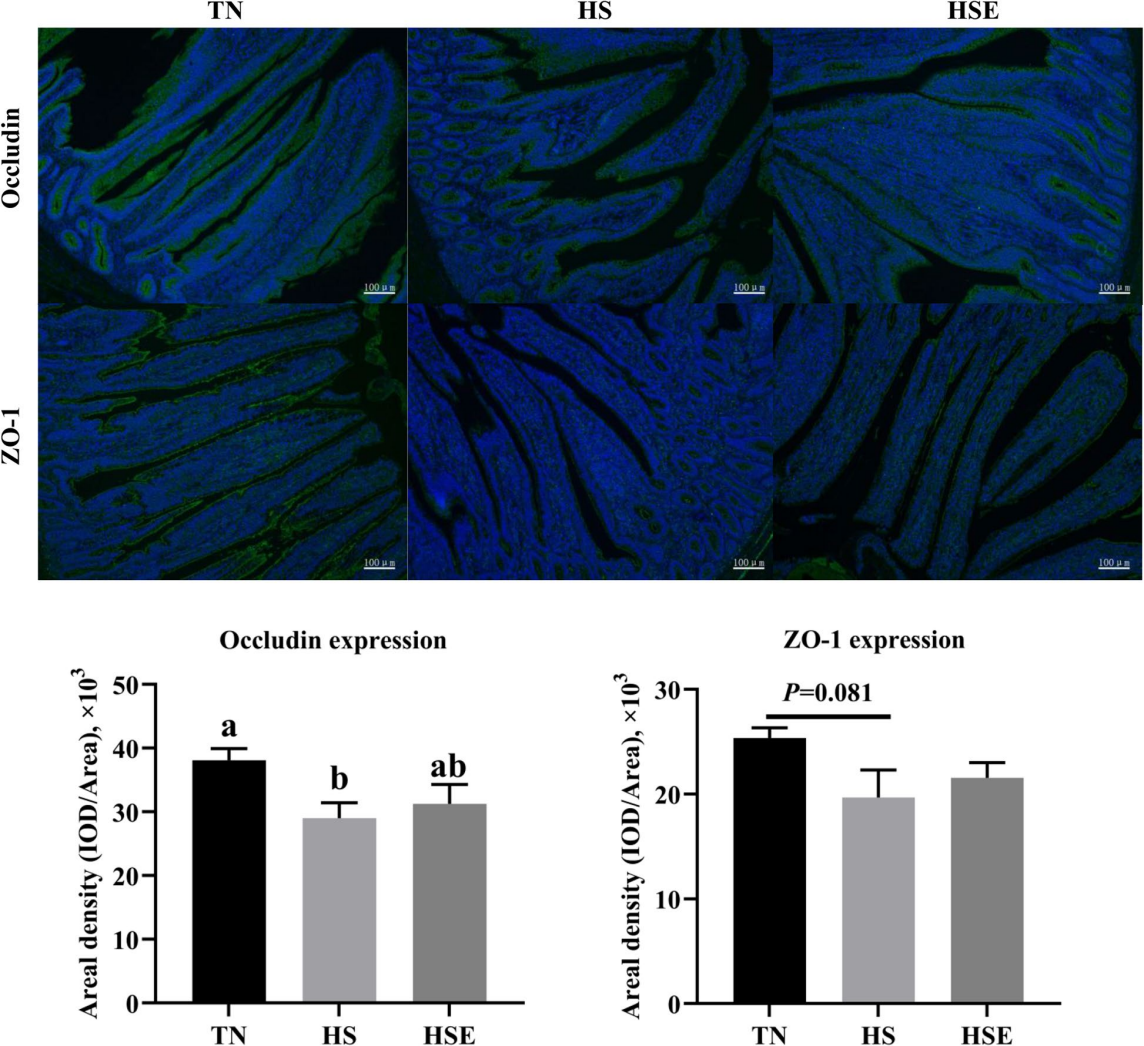
Immunofluorescence analysis of ileal tight junction proteins in yellow-feathered broilers under heat stress and supplemented with *Enteromorpha prolifera* polysaccharides. *TN* Thermal neutral zone, *HS* Heat stress, *HSE* Heat stress group supplemented 0.1% *Enteromorpha prolifera* polysaccharides. 100× magnification, the scale bars are 100 μm. ^a,b^Different superscripts indicate a significant difference between groups (*P* < 0.05)

According to the immunofluorescence analysis, HS reduced the Occludin and ZO-1 expression of jejunum compared with the TN group (Fig. 4, *P* < 0.05). However, dietary EPS improved the Occludin and ZO-1 expression of jejunum in heat-stressed broilers (*P* < 0.05). Compared with the TN group, broilers in the HS group had a lower expression level of Occludin (Fig. 5, *P* < 0.05); and tended to decrease the expression level of ZO-1 in the ileum (*P* = 0.081).

### Protein expression of the jejunum

**Fig. 6:**
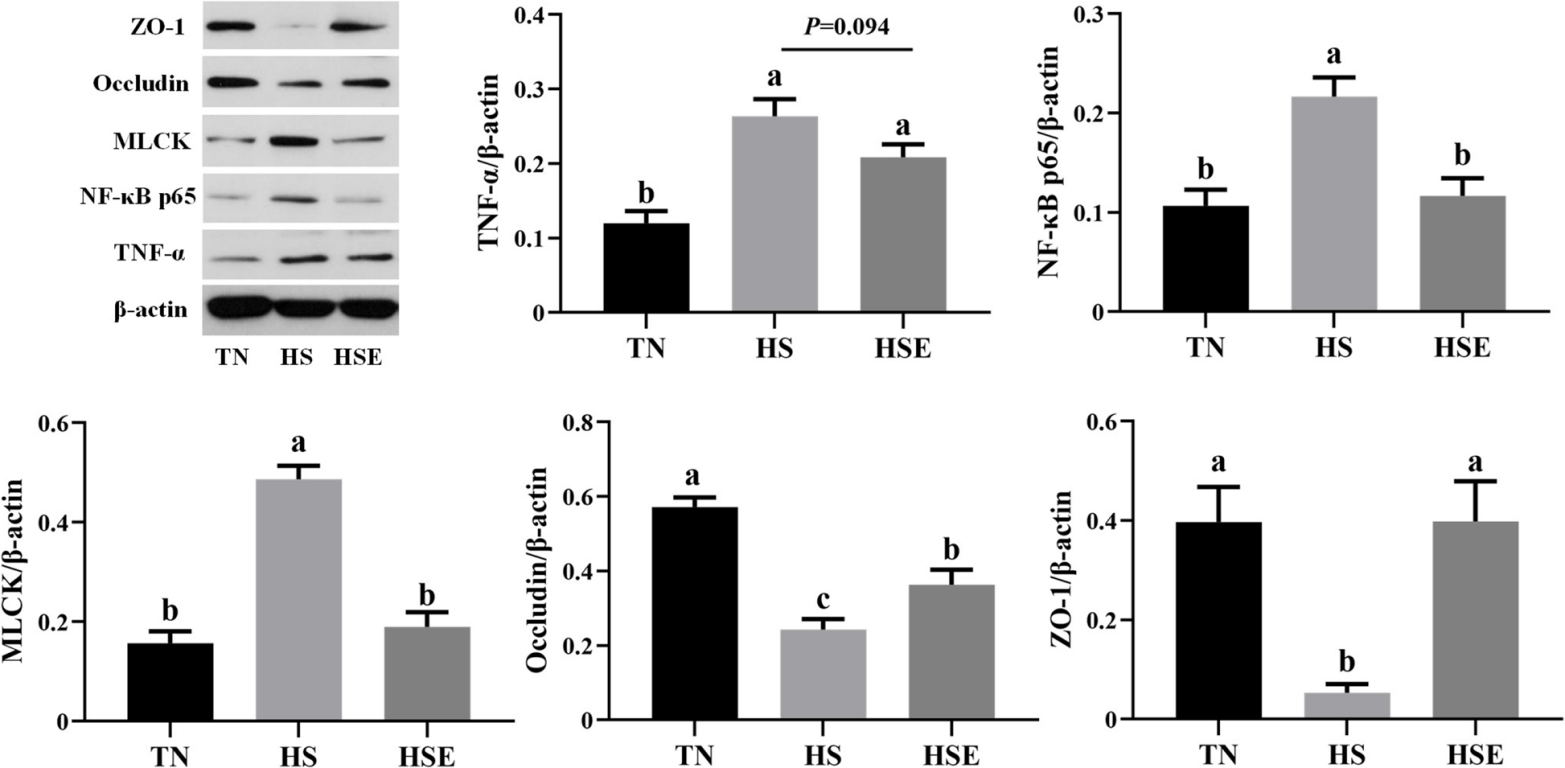
Effects of *Enteromorpha prolifera* polysaccharides on jejunal relative protein expression in yellow-feathered broilers under heat stress. *TN* Thermal neutral zone, *HS* Heat stress, *HSE* Heat stress group supplemented 0.1% *Enteromorpha prolifera* polysaccharides. ^a−c^Different superscripts indicate a significant difference between groups (*P* < 0.05)

Based on the fact that dietary EPS could alleviate the jejunum barrier function of heat-stressed broilers, we further investigated the protein expression of jejunum using Western blot (Fig. 6). The protein expression levels of NF-κB p65, TNF-α, and MLCK in jejunum were elevated by HS (*P* < 0.01). Conversely, the protein expression levels of Occludin and ZO-1 in jejunum were decreased by HS (*P* < 0.01). Dietary EPS reduced NF-κB p65 and MLCK protein expression (*P* < 0.01), tended to decrease the TNF-α protein expression (*P* = 0.094), and promoted Occludin and ZO-1 protein expression (*P* < 0.05) of the jejunum in broilers under HS.

### Cecal microbiota

**Fig. 7:**
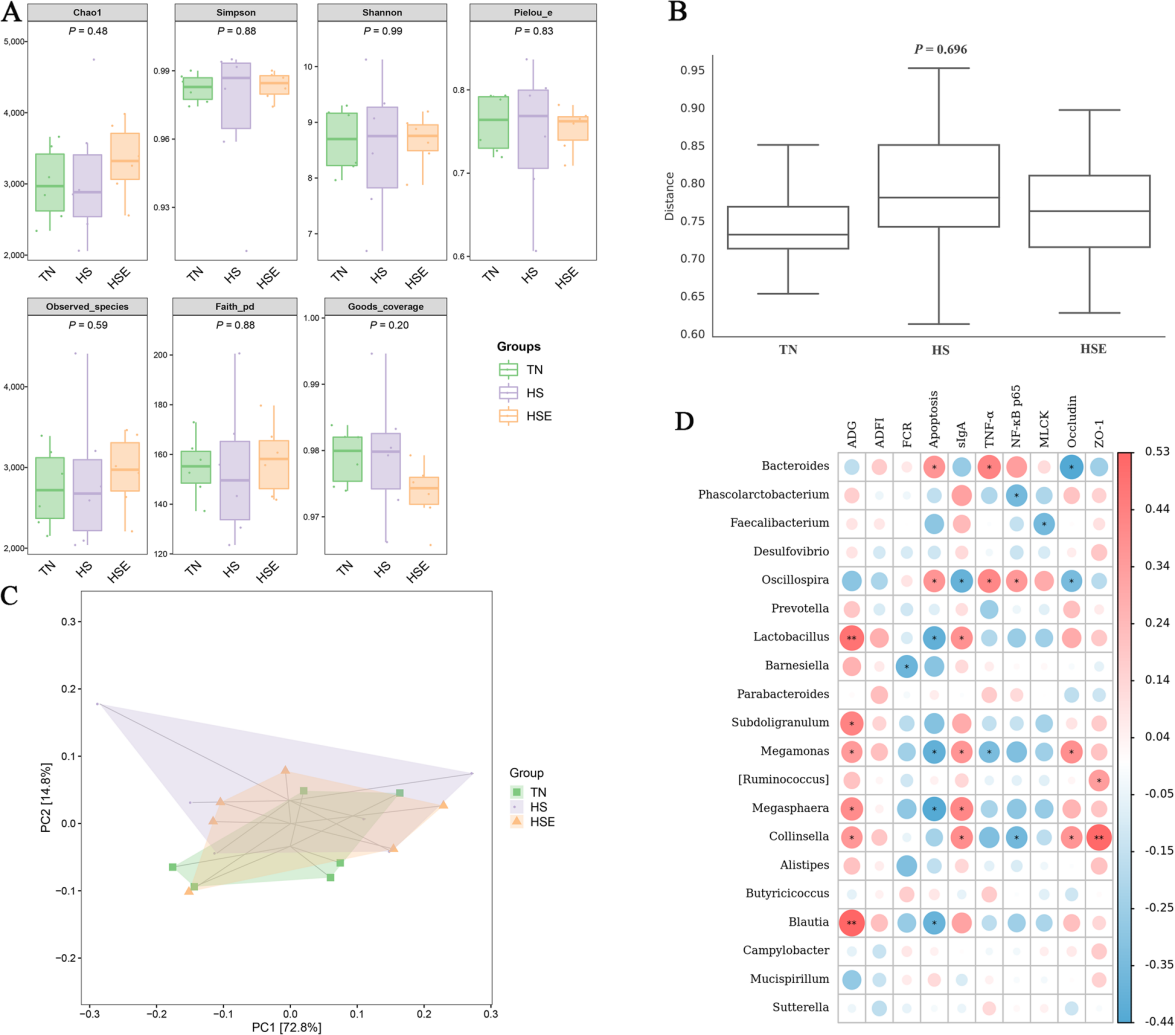
Effects of *Enteromorpha prolifera* polysaccharides on cecal microbiota in yellow-feathered broilers under heat stress. **A** Alpha diversity of cecal microbiota, **B** Detection of intergroup distance of cecal microbiota composition based on distance matrix analysis, **C** Principal component analysis (PCA) of cecal microbiota composition between groups, **D** Correlation analysis of cecal microbial proportions at the genus level with growth performance and jejunal barrier function among three groups, red represents a positive correlation, while blue represents a negative correlation. *TN* Thermal neutral zone, *HS* Heat stress, *HSE* Heat stress group supplemented 0.1% *Enteromorpha prolifera* polysaccharides. ^*^P < 0.05 and ^**^P < 0.01 indicate a significant correlation

HS treatment and dietary EPS supplementation had no significant impact on alpha diversity indexes (Chao 1, Simpson, Shannon, Pielou_e, Observed_species, Faith_pd, Goods_coverage) of cecal microbiota in broilers (Fig. 7A, *P *> 0.10). Distance matrix analysis suggests that there was no significant difference in the distance of microbiota composition between TN, HS, and HSE groups (Fig. 7B, *P *> 0.10). The PCA results show the differences in the total species composition of cecal microbiota between groups (Fig. 7C, PC1 = 72%, PC2 = 14.8%). OPLS-DA analysis indicates the differences in the species abundance composition of cecal microbiota between groups (Fig. S1, PC1 = 31.9%, PC2 = 22.4%). Table 5 shows the effects of HS treatment and EPS supplementation on the relative abundance of cecal microbiota at the phylum and genus levels. At the phylum level, there were no significant differences in the relative abundance of microbiota among the three groups (*P* > 0.10). At the genus level, HS decreased the relative abundance of *Megamonas* (*P* < 0.05); and broilers in the HSE group had no significant difference in the relative abundance of *Megamonas* compared with the TN group (*P* > 0.10). Figure 7D shows the correlation between cecal microbial proportions at the genus level and growth performance and jejunal barrier function among TN, HS and HSE groups. The proportion of *Bacteroides* had a positive correlation with jejunal apoptosis and TNF-α protein level (*P* < 0.05), and had a negative correlation with jejunal Occludin protein level (*P* < 0.05). The proportion of *Phascolarctobacterium* was negatively related to jejunal NF-κB p65 protein level (*P* < 0.05). The proportion of *Faecalibacterium* was negatively related to jejunal MLCK protein level (*P* < 0.05). The proportion of *Oscillospira* had a positive correlation with jejunal apoptosis, TNF-α and NF-κB p65 protein level (*P* < 0.05); and had a negative correlation with jejunal sIgA content and Occludin protein level (*P* < 0.05). The proportion of *Lactobacillus* was positively correlated with ADG (*P* < 0.01) and jejunal sIgA content (*P* < 0.05) and negatively correlated with jejunal apoptosis (*P* < 0.05). The proportion of *Barnesiella* was negatively related to FCR (*P* < 0.05). The proportion of *Subdoligranulum* was positively correlated with ADG (*P* < 0.05). The proportion of *Megamonas* had a positive correlation with ADG, jejunal sIgA content, and Occludin protein level (*P* < 0.05); and had a negative correlation with jejunal apoptosis and TNF-α protein level (*P* < 0.05). The proportion of *[Ruminococcus]* was positively correlated with jejunal ZO-1 protein level (*P* < 0.05). The proportion of *Megasphaera* had a positive association with ADG and jejunal sIgA content (*P* < 0.05) and had a negative correlation with jejunal apoptosis (*P* < 0.05). The proportion of *Collinsella* had a positive correlation with ADG, jejunal sIgA content, Occludin, and ZO-1 protein level (*P* < 0.05); and had a negative correlation with jejunal NF-κB p65 protein level (*P* < 0.05). The proportion of *Blautia* was positively related to ADG (*P* < 0.01) and was negatively related to jejunal apoptosis (*P* < 0.05).

**Table 5:** Effects of *Enteromorpha prolifera* polysaccharides on the relative abundance of cecal microbiota at the phylum (top 10) and genus (top 20) level in yellow-feathered broilers under heat stress

Items	TN	HS	HSE	SEM	*P*-value
Phylum					
Bacteroidetes	0.482	0.565	0.512	0.045	0.443
Firmicutes	0.402	0.342	0.374	0.036	0.529
Firmicutes/Bacteroidetes	0.887	0.645	0.790	0.146	0.522
Actinobacteria	0.031	0.011	0.012	0.010	0.335
Proteobacteria	0.051	0.037	0.051	0.011	0.588
Synergistetes	0.019	0.029	0.033	0.010	0.587
Tenericutes	0.0008	0.0005	0.0007	0.0003	0.703
Deferribacteres	0.0010	0.0008	0.0028	0.0007	0.108
TM7	0.0023	0.0028	0.0028	0.0010	0.962
Cyanobacteria	0.0002	0.0003	0.0003	0.0001	0.384
Verrucomicrobia	0.0001	0.0001	0.0003	0.00007	0.104
Genus					
*Bacteroides*	0.210	0.249	0.230	0.061	0.907
*Phascolarctobacterium*	0.103	0.088	0.093	0.019	0.842
*Faecalibacterium*	0.060	0.039	0.053	0.019	0.719
*Desulfovibrio*	0.043	0.029	0.042	0.010	0.544
*Oscillospira*	0.015	0.034	0.024	0.007	0.165
*Prevotella*	0.022	0.023	0.017	0.007	0.786
*Lactobacillus*	0.018	0.010	0.013	0.005	0.673
*Barnesiella*	0.014	0.011	0.012	0.004	0.928
*Parabacteroides*	0.013	0.011	0.012	0.003	0.937
*Subdoligranulum*	0.008	0.004	0.015	0.005	0.295
*Megamonas*	0.020^a^	0.002^b^	0.005^ab^	0.005	0.097
*[Ruminococcus]*	0.009	0.008	0.009	0.002	0.828
*Megasphaera*	0.009	0.004	0.007	0.004	0.622
*Collinsella*	0.0072	0.0005	0.0015	0.004	0.383
*Alistipes*	0.003	0.002	0.003	0.0005	0.272
*Butyricicoccus*	0.0026	0.0027	0.0025	0.0005	0.982
*Blautia*	0.0037	0.0015	0.0022	0.0008	0.175
*Campylobacter*	0.0018	0.0008	0.0021	0.0009	0.594
*Mucispirillum*	0.0010	0.0008	0.0028	0.0007	0.108
*Sutterella*	0.0008	0.0012	0.0018	0.0004	0.263

*TN* Thermal neutral zone, *HS* Heat stress, *HSE* Heat stress group supplemented 0.1% *Enteromorpha prolifera* polysaccharides, *SEM* Standard error of the mean, *P P* value for analysis of variance (ANOVA)

^a,b^Different superscripts in the same row indicate a significant difference between groups (*P* < 0.05)

## Discussion

### Growth performance

In this study, we found that dietary supplementation with EPS increased the ADG of broilers aged 3 to 4 weeks under HS conditions. Several studies have confirmed the growth-stimulating benefits of EPS in broilers [9–11]. Furthermore, Akinyemi et al. [22] and Archer et al. [23] suggested that dietary brown seaweed (*Ascophyllum nodosum*) extracts improve the ADG and FCR in broilers under HS. Wang et al. [24] found a significant improvement in the body weight of heat-stressed broilers after feeding the polysaccharides from *Abrus cantoniensis.*

### Intestinal histomorphology

Intestinal health plays a crucial role in broiler production. HS causes a decline in growth rate can be attributed to the impairment of the intestinal barrier function of broilers [3]. Intestinal histomorphology is an important indicator of intestinal barrier function and is associated with nutrient absorption. Previous studies have shown that EPS supplementation resulted in a longer villus height of the ileum and jejunum, suggesting the beneficial effects of EPS on improving intestinal histomorphology in broilers [9, 10]. However, the present study failed to obtain a significant effect of dietary EPS on protecting the histomorphology of ileum and jejunum in heat-stressed broilers. When broilers are raised in a thermal environment, the peripheral blood flow is accelerated to increase heat dissipation, leading to insufficient blood supply to the intestines, which affects the proliferation of intestinal epithelial cells and suppresses the renewal and growth of intestinal epithelial villi [25]. Under the physiological status of intestinal ischemia, restoring intestinal histomorphology to the level of broilers reared in thermoneutral conditions is difficult by using only nutritional intervention strategies [1]. This may explain the weak effect of EPS in alleviating HS-induced intestinal histo-morphological damage observed in this study.

### Intestinal antioxidant and sIgA content

It is well known that HS not only induces intestinal ischemia but also reduces intestinal mucosal antioxidant performance and immunity [26, 27]. Likewise, the antioxidant capacity and the secretion of sIgA in the ileum and jejunum of broilers were decreased by HS in the present study, which are in agreement with previous findings [28–30]. However, dietary EPS reversed the decrease in the activity of T-SOD and GST and the level of sIgA in the jejunal mucosa of broilers under HS. Consistently, in vitro experiments have shown that EPS has excellent antioxidant function [6, 31]. An in vivo study by Liu et al. [9] demonstrated that dietary EPS could improve the T-SOD and GST activity of jejunum in broilers. Wang et al. [24] observed that supplementation of natural polysaccharides from *Abrus cantoniensis* enhanced the serum SOD activity in broilers under HS. In addition, previous studies found that plant-derived polysaccharides could stimulate the sIgA generation of the small intestine against pathogenic microbial infections in broilers [32, 33]. Studies have confirmed that HS could result in the dysfunction of antioxidant defense systems through overproducing reactive oxygen species (ROS) and reducing antioxidant enzyme activity [27]. The excessive ROS caused by HS leads to widespread damage to proteins and nucleic acids of intestinal epithelial cells, thereby disrupting the intestinal barrier function [3, 4]. The antioxidant enzymes, including SOD and GST, can help to protect intestinal epithelial cells against ROS-induced damage [34]. Furthermore, the immune antibody sIgA is the first line of defending the digestive tract from antigens and pathogens invasion by preventing their contact with epithelial cell receptors [35]. The production of sIgA is involved in the balance of pro-inflammatory and anti-inflammatory cytokines. Broilers exposed to HS have impaired intestinal integrity and increased permeability to endotoxins and microorganisms, thus prompting gut inflammatory reactions and destroying the equilibrium state of cytokines, ultimately inhibiting the intestinal sIgA synthesis [28, 29]. Therefore, in this study, dietary EPS could improve the antioxidant capacity and immunity of the jejunal mucosa by enhancing the activity of T-SOD and GST and the production of sIgA, which directly contributed to the intestinal barrier function of broilers challenged with HS.

### Apoptosis of intestinal epithelial cells

As the main components of the gut mechanical barrier, the intestinal epithelial cells are mainly accountable for regulating intestinal integrity and permeability. Concerning the apoptosis of intestinal epithelial cells, the current study indicated that dietary EPS mitigated HS-induced apoptosis of ileum and jejunum in broilers. Similar to our findings, Liang et al. [36] and Li et al. [37] also reported that the polysaccharides extracted from the edible mushroom *Ganoderma lucidum* could reduce the apoptosis caused by toxins in mice and intestinal epithelial cell lines. According to Ajala et al. [38], H_2_O_2_-caused intestinal epithelial cell apoptosis could be ameliorated by red alga *Gelidium spinosum* polysaccharides, and the above two plant polysaccharides and EPS have similar monosaccharides composition and molecular weight. They stated that this beneficial effect was partially attributed to the elevated SOD activity and antioxidant function of natural polysaccharides. However, there are few available studies regarding the effects of EPS on intestinal apoptosis in broilers, especially under HS conditions. Therefore, additional investigations are required to illustrate the specific mechanisms.

### Intestinal tight junction function

Moreover, the paracellular permeability of intestinal epithelial cells depends on tight junction proteins. Nevertheless, the expression of tight junction proteins is vulnerable to external stressors, such as HS [25]. Numerous studies have demonstrated that HS reduced the expression of intestinal tight junction proteins, including Zonula occludens (ZOs), Occludin, and Claudins [39–41]. Consistent with relevant research, heat-stressed broilers showed a lower gene and protein expression level of jejunal Occludin and ZO-1 in this study. Interestingly, dietary EPS promoted Occludin and ZO-1 expression in the jejunal mucosa of broilers under HS, as characterized by mRNA, immunofluorescence, and Western blot. In agreement, EPS has been reported to enhance the expression of Occludin in the jejunal mucosa of broilers [10]. Liu et al. [13] found that dietary seaweed polysaccharides improved the duodenal Occludin and ZO-1 expression in broilers during HS. Natural polysaccharides from other medicinal plants also exhibit the ability to alleviate the decrease in intestinal tight junction proteins expression induced by HS in broilers [24, 42, 43].

MLCK is a key molecule in modulating intestinal tight junctions. MLCK mainly regulates the contraction of the epithelial cytoskeleton; under the stimulation of various stressors, the expression of MLCK increases, causing the contraction and remodeling of the cytoskeleton, resulting in the shedding of tight junctions between epithelial cells and reducing the expression of tight junctions [44]. Emerging evidence indicates that the inhibition of MLCK leads to an elevation in gut tight junction protein expression [45]. In the present study, HS increased the mRNA and protein expression of MLCK in the jejunal mucosa of broilers, suggesting that HS-induced tight junction disruption may be related to the increased expression of MLCK. In particular, the jejunal protein level of MLCK was suppressed in heat-exposed broilers after feeding an EPS-containing diet. Similarly, it has been shown that natural polysaccharides upregulated tight junction protein’s expression by inhibiting the activation of MLCK in mice and caco-2 cells [46, 47]. Therefore, EPS promoted the tight junction function of jejunal epithelial cells in heat-stressed broilers, probably through regulating MLCK signaling.

### Nrf2 and NF-κB signaling pathways

Evidence suggests that HS-induced intestinal barrier damage is closely related to oxidative stress and inflammatory response [4]. To further investigate the potential molecular mechanisms of EPS improving jejunal barrier function in heat-stressed broilers, this study evaluated the regulatory effect of EPS on the Nrf2 and NF-κB signaling pathways. Nrf2 is a nuclear transcription factor with a leucine zipper structure and belongs to Cap'n'collar (CNC) transcription factor family [48]. It has generally been known that the key role of Nrf2 pathway is in regulating the transcription of antioxidant enzyme genes and preventing oxidative stress [49]. Although this study found that dietary EPS enhanced the activity of T-SOD and GST and the mRNA level of *γ-GCLc* in jejunal mucosa of heat-stressed broilers, HS and EPS have no significant effect on *Nrf2* mRNA expression. Wang et al. [24] also observed that dietary plant-derived polysaccharides could improve the SOD activity but had no significant influence on Nrf2 protein level of serum and liver in broilers during HS. On the contrary, previous studies reported that dietary EPS upregulated the mRNA expression of antioxidant-related genes of the spleen and duodenum in heat-stressed broilers by activating Nrf2 signaling [13, 14]. These findings indicate that the regulation of Nrf2 signaling pathway by EPS may have tissue specificity. Meanwhile, the improvement of antioxidant enzymes activity may be associated with the structural interaction between EPS molecules and enzymes rather than modulating Nrf2 signaling [8].

In addition, the inflammatory response caused by HS is widely recognized as a key trigger for the impairment of intestinal barrier integrity [27]. NF-κB is a classic signaling pathway that regulates the inflammatory response. HS induces the activation of NF-κB and promotes the release of pro-inflammatory cytokines, disrupting the equilibrium condition between anti-inflammatory and pro-inflammatory cytokines and leading to intestinal barrier injury [3, 4]. Notably, dietary EPS could reduce the mRNA and protein level of NF-κB p65, downregulate the mRNA expression of pro-inflammatory cytokines *TNF-α* and *IL-1β* and promote the mRNA level of anti-inflammatory cytokine *IL-10* in the jejunal mucosa of broilers under HS. These suggest that EPS suppressed the activation of NF-κB-mediated inflammatory response signaling pathway, contributing to relieving the intestinal inflammatory damage caused by HS. This is consistent with the available literatures, which showed that EPS supplementation alleviated HS-induced inflammatory response in duodenum and immune organs of broilers by downregulating NF-κB p65 expression [12–14]. Also, other plant-based polysaccharides have been reported to reduce gut pro-inflammatory cytokines level but increase anti-inflammatory cytokines content through suppressing NF-κB p65 activation in broilers challenge with lipopolysaccharide [50, 51]. It is worth noting that, there is an interaction between inflammatory reactions and the integrity of the intestinal mechanical barrier. Inhibiting the NF-κB p65-mediated inflammatory signaling pathway can reduce intestinal epithelial cell apoptosis [52, 53] and improve tight junction protein expression [13, 54]. Specifically, inflammatory damage can be directly manifested in an elevation of apoptosis rate; and the activation of NF-κB p65 promotes the expression of MLCK, thereby reducing the expression of intestinal tight junction protein [55, 56]. Several studies have confirmed that the polysaccharides from various plants increased the expression of the tight junctions by downregulating NF-κB/MLCK signaling pathway in mice and/or caco-2 cell models [57–59]. Thus, dietary EPS preserved intestinal integrity in broilers during HS might by suppressing the NF-κB/MLCK signaling pathway activation. Further verifications are necessary, such as co-immunoprecipitation of NF-κB p65 and MLCK.

### Cecal microbiota

Maintaining gut microbiota homeostasis is crucial for the intestinal health of broilers. It has been suggested that HS-induced adverse impacts on intestinal barrier function were accompanied by alterations in cecal microbiota composition [2]; and a large number of reports have demonstrated that nutritional intervention could improve intestinal barrier integrity by modulating the cecal microbiota in broilers exposed to different stressors [24, 54]. In the current study, there were no significant differences in alpha diversity indexes of cecal microbiota in broilers with HS and EPS treatment. Consistently, Liu et al. [2] reported that HS did not affect the alpha diversity of cecal microbiota in broilers. Also, Wang et al. [24] found that HS and dietary natural polysaccharides had no significant effects on the alpha diversity of cecal microbiota in broilers. Additionally, even though HS and EPS treatment had weak influence on the relative abundance of each bacteria in the cecum, the proportion of certain bacteria at the genus level among the three groups was found to be significantly correlated with growth performance and intestinal barrier function in this study. Wherein the proportion of *Bacteroides* and *Oscillospira* among three groups showed a negative correlation with intestinal barrier function. *Bacteroides* is a Gram-negative bacterium that normally inhabits the intestines of animals. The capsule polysaccharides produced by some species of *Bacteroides* (such as *Bacteroides fragilis*) are common intestinal pathogenic factors that can cause intestinal inflammation [60]. Qiao et al. [61] also observed that the polysaccharides derived from *Astragalus membranaceus* and *Glycyrrhiza uralensis* reduced cecal relative abundance of *Bacteroides*, and it was positively correlated with the content of pro-inflammatory cytokines in the intestine of broilers. *Oscillospira* is a widely present bacterium in animal intestines that has not been successfully cultured in vitro; in the mouse model, the abundance of *Oscillospira* was also found to be moderately reduced by dietary marine polysaccharides, and *Oscillospira* was negatively correlated with intestinal tight junction but positively correlated with pro-inflammatory cytokines [62, 63]. Meanwhile, the proportion of *Phascolarctobacterium, Faecalibacterium, Lactobacillus, Barnesiella, Subdoligranulum, Megamonas, [Ruminococcus], Megasphaera, Collinsella,* and *Blautia* between groups exhibited a positive correlation with growth performance or intestinal barrier function. *Phascolarctobacterium* has been proven to be able to utilize polysaccharides to produce short chain fatty acids (SCFAs), including acetic acid and propionic acid, promoting intestinal health [64]. *Faecalibacterium* belongs to the Firmicutes phylum and is an important producer of butyric acid. It has been reported as a potential probiotic in the gut and exerts anti-inflammatory effects by inhibiting the activation of NF-κB p65 [65]. *Lactobacillus* is widely used as a probiotic, and its growth in the intestine can be stimulated by dietary polysaccharides, which is beneficial for intestinal barrier integrity in animals [66]. Recent clues indicated that *Subdoliganulum* promoted IgA secretion and enhanced intestinal immunity [67]. *Megamonas* and *Megasphaera* can ferment carbohydrates to produce SCFAs, such as acetic acid and propionic acid, contributing to host gut health [68]. *[Ruminococcus]* ferments fibers to produce formic acid and acetic acid, which is generally believed to promote intestinal health [69]. Besides, *Barnesiella, Collinsella,* and *Blautia* have also been reported to act as new functional genus with potential probiotic properties to alleviate intestinal inflammation [70, 71]. Therefore, dietary EPS may improve the intestinal barrier function of heat-stressed broilers by modulating the proportion of beneficial gut bacteria. However, additional research is needed to establish the causal relationship between microbiota and intestinal barrier function.

## Conclusions

Dietary EPS improved the growth performance of broilers under HS, and dietary EPS ameliorated HS-induced impairment of intestinal barrier integrity by suppressing the activation of NF-κB/MLCK signaling pathway, and these beneficial effects are also related to the cecal microbial community. The present findings indicate that EPS can serve as a potential anti-HS agent to improve growth performance and gut health in yellow-feathered broilers, simultaneously contributing to developing nutritional strategies to mitigate HS in animals.

### Supplementary Information

**Additional file 1: Table S1. **The chemical and monosaccharide composition of seaweed-derived polysaccharides (SDP) from Enteromorpha prolifera.** Table S2.** The formulation and nutrient level of the basal diets. Table S3. Primer information of real-time quantitative PCR.

**Additional file 2: Fig. S1. **OPLS-DA (orthogonal partial least squares discriminant analysis) analysis of cecal microbiota composition. *TN* Thermal neutral zone, *HS* Heat stress, *HSE* Heat stress group supplemented 0.1% Enteromorpha prolifera polysaccharides.

## Data Availability

All datasets generated for this study are available from the first and corresponding authors upon reasonable request.

## Abbreviations

ADG: Average daily gain
ADFI: Average daily feed intake
CAT1: Catalase1
CD: Crypt depth
EPS: *Enteromorpha prolifera* polysaccharides
FCR: Feed conversion rate (the ratio of feed/gain)
GPx1: Glutathione peroxidase 1
GPx3: Glutathione peroxidase 3
HO-1: Heme oxygenase 1
HS: Heat stress
HSE: Heat stress group supplemented 0.1% EPS
IFN-γ: Interferon-γ
IL-1β: Interleukin 1β
IL-2: Interleukin 2
IL-4: Interleukin 4
IL-6: Interleukin 6
IL-10: Interleukin 10
MLCK: Myosinlightchainkinase
NF-κB p65: Nuclear factor-kappaB p65
Nrf2: Nuclear factor-erythroid2-related factor 2
OPLS-DA: Orthogonal partial least squares discriminant analysis
PCA: Principal component analysis
SEM: Standard error of the mean
sIgA: Secretory immunoglobulin A
SOD1: Superoxide dismutase 1
SOD2: Superoxide dismutase 2
TLR4: Toll-like receptor 4
TN: Thermal neutral zone
TNF-α: Tumor necrosis factor-α
VH: Villus height
VW: Villus width
VH:CD: The ratio of villus height/crypt depth
ZO-1: Zonula occludens-1
γ-GCLc: γ-Glutamylcysteine ligase catalytic subunit
γ-GCLm: γ-Glutamate-cysteine ligase modifier subunit
