# Supplementary material for: Effects of Enteromorpha prolifera polysaccharides on growth performance, intestinal barrier function and cecal microbiota in yellow-feathered broilers under heat stress
Source: J Anim Sci Biotechnol. 2023 Oct 10;14:132. doi: 10.1186/s40104-023-00932-2 (PMC10563363; doi:10.1186/s40104-023-00932-2)
Supplement: Supplementary file 1 — Additional file 1: Table S1. The chemical and monosaccharide composition of seaweed-derived polysaccharides (SDP) from Enteromorpha prolifera. Table S2. The formulation and nutrient level of the basal diets. Table S3. Primer information of real-time quantitative PCR. [file 40104_2023_932_MOESM1_ESM.docx]

| **Table S1** The chemical and monosaccharide composition of seaweed-derived polysaccharides (SDP) from *Enteromorpha prolifera*. | |
| --- | --- |
| **Items** | **Contents, %** |
| **Chemical composition** |  |
| Total polysaccharides | 53.32 |
| Protein | 1.65 |
| Sulfate | 19.87 |
| Uronic acid | 12.66 |
| Moisture | 3.58 |
| Unspecified compounds (lipid, salt, etc.) | 8.92 |
| **Monosaccharide composition** |  |
| Glucosamine (GlcN) | 50.81 |
| Glucose (Glc) | 27.70 |
| Galacturonic acid (GalA) | 11.75 |
| Mannose (Man) | 2.78 |
| Xylose (Xyl) | 2.63 |
| Galactose (Gal) | 1.95 |
| Arabinose (Ara) | 0.93 |
| Glucuronic acid (GlcA) | 0.76 |
| Fucose (Fuc) | 0.27 |
| Galactosamine (GalN) | 0.25 |
| Ribose (Rib) | 0.17 |
| A phenol‑sulfuric acid assay was used to determine the total polysaccharide content and Glc was used as a standard. Bradford's method was used to analysis the protein content and bovine serum albumin (BSA) was used as a standard. The barium sulfate-gelatin turbidimetric method was used to determine the sulfate content and potassium sulfate was used as a standard. m-Hydroxybiphenyl colorimetry was used to measure uronic acid content and d-GlcA was used as a standard. Monosaccharide composition was determined using high-performance liquid chromatography (HPLC). | |

| **Table S2** The formulation and nutrient level of the basal diets. | |
| --- | --- |
| **Item** | **Contents (%)** |
| **Ingredients** |  |
| Corn | 60.84 |
| Soybean meal | 32.11 |
| Wheat bran | 2.16 |
| Soybean oil | 2.00 |
| Limestone | 1.28 |
| CaHPO_4_ | 1.26 |
| _DL_-Methionine | 0.15 |
| Vitamin premix^1^ | 0.10 |
| Mineral premix^2^ | 0.10 |
| Total | 100.00 |
| **Nutrient level^3^** |  |
| ME (MJ/kg) | 11.94 |
| Crude protein (%) | 18.22 |
| Ca (%) | 0.98 |
| Met (%) | 0.32 |
| Cystine (%) | 0.31 |
| Lys (%) | 0.90 |
| Total phosphorus (%) | 0.51 |
| ^1^Premix provided per kilogram of diet: 5,000 IU of vitamin A, 1000 IU of vitamin D_3_, 10 IU of vitamin E, 0.5 mg of vitamin K_3_, 3 mg of thiamin, 7.5 mg of riboflavin, 4.5 mg of vitamin B_6_, 10 μg of vitamin B_12_, 25 mg of niacin, 0.55 mg of folic acid, 0.2 mg of biotin, 500 mg of choline, and 10.5 mg of pantothenic acid.  ^2^Premix provided per kilogram of diet: 60 mg of Zn, 80 mg of Mn, 80 mg of Fe, 3.75 mg of Cu, 0.35 mg of I, and 0.15 mg of Se.  ^3^Except for metabolic energy (ME), others are measured values. | |

| **Table S3** Primers information of real-time quantitative PCR. | | | |
| --- | --- | --- | --- |
| Target Genes | Primer | Primer Sequence (5'→3') | Accession No. |
| *MLCK* | Forward | TGCTACATCCTGGTCAGCG | NM_001322361.1 |
|  | Reverse | GATAAAGTCCTTGGCATCGTC |  |
| *Cadherin* | Forward | GACAGGGACATGAGGCAGAA | NM_001039258.2 |
|  | Reverse | GCCGTGACAATGCCATTCTC |  |
| *Occludin* | Forward | CTGCTGTCTGTGGGTTCCT | NM_205128.1 |
|  | Reverse | CCAGTAGATGTTGGCTTTGC |  |
| *Claudin-1* | Forward | ATGACCAGGTGAAGAAGATGC | NM_001013611.2 |
|  | Reverse | TGCCCAGCCAATGAAGAG |  |
| *Claudin-4* | Forward | GAAGCGCTGAACCGATACCA | XM_003642382.6 |
|  | Reverse | TGCTTCTGTGCCTCAGTTTC |  |
| *ZO-1* | Forward | CGTAGTTCTGGCATTATTCGT | XM_015278981.2 |
|  | Reverse | TGGGCACAGCCTCATTCT |  |
| *Mucin-2* | Forward | TGAGTCAGGCATAAATCGTGT | NM_001318434.1 |
|  | Reverse | CAGGTCTAAGTCGGGAAGTGTA |  |
| *Nrf2* | Forward | TGTGTGTGATTCAACCCGACT | NM_205117.1 |
|  | Reverse | TTAATGGAAGCCGCACCACT |  |
| *HO-1* | Forward | TTGGCAAGAAGCATCCAGA | NM_205344.1 |
|  | Reverse | TCCATCTCAAGGGCATTCA |  |
| *SOD1* | Forward | TTGTCTGATGGAGA TCATGGCTTC | NM_205064.1 |
|  | Reverse | TGCTTGCCTTCAGGATTAAAGTGAG |  |
| *SOD2* | Forward | CAGATAGCAGCCTGTGCAAATCA | NM_204211.1 |
|  | Reverse | GCATGTTCCCATACATCGATTCC |  |
| *GPx1* | Forward | GACCAACCCGCAGTACATCA | NM_001277853.1 |
|  | Reverse | GAGGTGCGGGCTTTCCTTTA |  |
| *GPx3* | Forward | CCTGCAGTACCTCGAACTGA | NM_001163232 |
|  | Reverse | CTTCAGTGCAGGGAGGATCT |  |
| *CAT1* | Forward | ACCAAGTACTGCAAGGCGAAAGT | XM_015277937.2 |
|  | Reverse | ACCCAGATTCTCCAGCAACAGTG |  |
| *γ-GCLc* | Forward | TGCGGTTCTGCACAAAATGG | XM_419,910.3 |
|  | Reverse | TGCTGTGCGATGAATTCCCT |  |
| *γ-GCLm* | Forward | CCAGAACGTCAAAGCACACG | NM_0,010,07953.1 |
|  | Reverse | TCCTCCCATCCCCCAGAAAT |  |
| *TLR4* | Forward | TGAAAGAGCTGGTGGAACCC | NM_001030693.1 |
|  | Reverse | CCAGGACCGAGCAATGTCAA |  |
| *NF-κB p65* | Forward | GTGTGAAGAAACGGGAACTG | NM_205129 |
|  | Reverse | GGCACGGTTGTCATAGATGG |  |
| *TNF-α* | Forward | GCCTATGCCAACAAGTACACCT | NM_204267.1 |
|  | Reverse | GCCAAGTCAACGCTCCTG |  |
| *IFN-γ* | Forward | CTTCCTGATGGCGTGAAGA | NM_205149.1 |
|  | Reverse | GAGGATCCACCAGCTTCTGT |  |
| *IL-1β* | Forward | CGCCGCTACCAGAGGGACTT | NM_204524.1 |
|  | Reverse | CCGGACCCAGTTGACCCCAT |  |
| *IL-2* | Forward | ATCTTTGGCTGTATTTCGGTAG | NM_204153.1 |
|  | Reverse | TCCTGGGTCTCAGTTGGTG |  |
| *IL-4* | Forward | CTCCTCACTGCCCACCCT | NM_001007079.1 |
|  | Reverse | CATCTTGACGCAGGAAACCT |  |
| *IL-6* | Forward | GATCCGGCAGATGGTGATAA | NM_204628.1 |
|  | Reverse | AGGATGAGGTGCATGGTGAT |  |
| *IL-10* | Forward | GCTCTCCTTCCACCGAAACC | NM_001004414.2 |
|  | Reverse | GGAGCAAAGCCATCAAGCAG |  |
| *β-actin* | Forward | TCAGGGTGTGATGGTTGGTATG | NM_205518.1 |
|  | Reverse | TGTTCAATGGGGTACTTCAGGG |  |
