# Supplementary figures and images for: Effects of Enteromorpha prolifera polysaccharides on growth performance, intestinal barrier function and cecal microbiota in yellow-feathered broilers under heat stress
Source: J Anim Sci Biotechnol. 2023 Oct 10;14:132. doi: 10.1186/s40104-023-00932-2 (PMC10563363; doi:10.1186/s40104-023-00932-2)

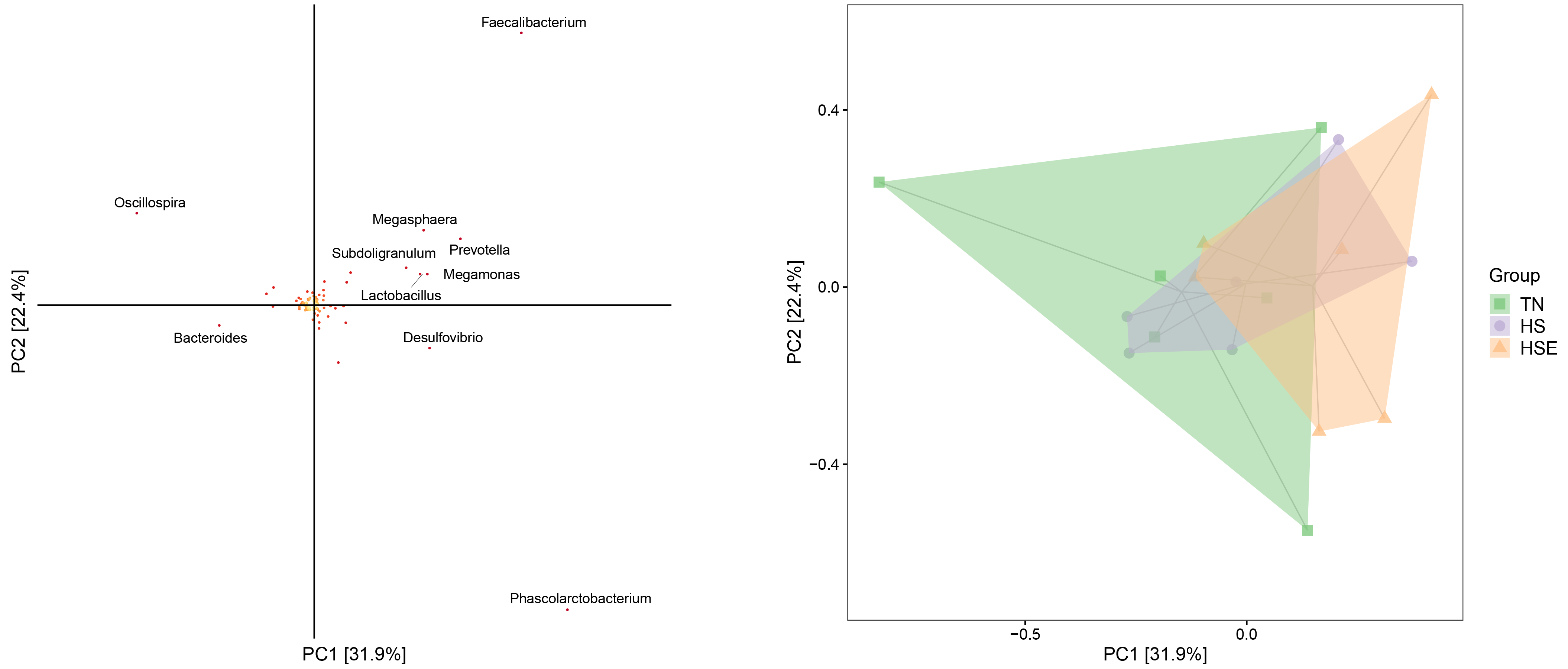

Supplement: Supplementary file 2 — Additional file 2: Fig. S1. OPLS-DA (orthogonal partial least squares discriminant analysis) analysis of cecal microbiota composition. TN Thermal neutral zone, HS Heat stress, HSE Heat stress group supplemented 0.1% Enteromorpha prolifera polysaccharides. [file 40104_2023_932_MOESM2_ESM.tif]
